# Chemoresistant ovarian cancer enhances its migration abilities by increasing store-operated Ca^2+^ entry-mediated turnover of focal adhesions

**DOI:** 10.1186/s12929-020-00630-5

**Published:** 2020-02-21

**Authors:** Ho-Kai Huang, Yi-Hsin Lin, Heng-Ai Chang, Yi-Shyun Lai, Ying-Chi Chen, Soon-Cen Huang, Cheng-Yang Chou, Wen-Tai Chiu

**Affiliations:** 1 Department of Biomedical Engineering, National Cheng Kung University, Tainan, 701 Taiwan; 2 Institute of Basic Medical Sciences, National Cheng Kung University, Tainan, 701 Taiwan; 3 Department of Obstetrics and Gynecology, Chi Mei Medical Center, Liouying Campus, Tainan, 736 Taiwan; 4 Department of Obstetrics and Gynecology, National Cheng Kung University, Tainan, 701 Taiwan; 5 Medical Device Innovation Center, National Cheng Kung University, Tainan, 701 Taiwan

**Keywords:** Chemoresistance, Ovarian cancer, Cell migration, Focal adhesions, Store-operated Ca^2+^ entry

## Abstract

**Background:**

Among gynecological cancers, ovarian carcinoma has the highest mortality rate, and chemoresistance is highly prevalent in this cancer. Therefore, novel strategies are required to improve its poor prognosis. Formation and disassembly of focal adhesions are regulated dynamically during cell migration, which plays an essential role in cancer metastasis. Metastasis is intricately linked with resistance to chemotherapy, but the molecular basis for this link is unknown.

**Methods:**

Transwell migration and wound healing migration assays were used to analyze the migration ability of ovarian cancer cells. Real-time recordings by total internal reflection fluorescence microscope (TIRFM) were performed to assess the turnover of focal adhesions with fluorescence protein-tagged focal adhesion molecules. SOCE inhibitors were used to verify the effects of SOCE on focal adhesion dynamics, cell migration, and chemoresistance in chemoresistant cells.

**Results:**

We found that mesenchymal-like chemoresistant IGROV1 ovarian cancer cells have higher migration properties because of their rapid regulation of focal adhesion dynamics through FAK, paxillin, vinculin, and talin. Focal adhesions in chemoresistant cells, they were smaller and exhibited strong adhesive force, which caused the cells to migrate rapidly. Store-operated Ca^2+^ entry (SOCE) regulates focal adhesion turnover, and cell polarization and migration. Herein, we compared SOCE upregulation in chemoresistant ovarian cancer cells to its parental cells. SOCE inhibitors attenuated the assembly and disassembly of focal adhesions significantly. Results of wound healing and transwell assays revealed that SOCE inhibitors decreased chemoresistant cell migration. Additionally, SOCE inhibitors combined with chemotherapeutic drugs could reverse ovarian cancer drug resistance.

**Conclusion:**

Our findings describe the role of SOCE in chemoresistance-mediated focal adhesion turnover, cell migration, and viability. Consequently, SOCE might be a promising therapeutic target in epithelial ovarian cancer.

**Graphical abstract:**

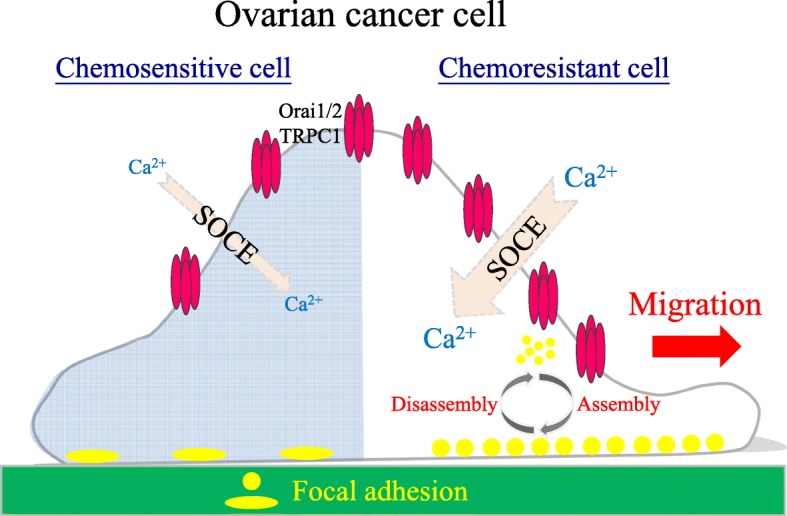

## Background

Ovarian cancer is the second most common gynecological malignancy in women, and it is relatively rare compared to other types of cancer; its mortality rate ranges from 2.2 to 7.1% of all cancer deaths [1, 2]. However, over 50% of patients diagnosed with ovarian cancer eventually die, making it the leading cause of death among all gynecological cancers [3]. The high fatality of ovarian cancer is due to its unapparent signs and symptoms before cancer cells spread outside the ovary, rendering it difficult to be detected at an early stage. Hence, ovarian cancer is typically not diagnosed until the late-stages (stages III and IV), and this makes its five-year relative survival rate extremely low (approximately 35 and 15% for stages III and IV, respectively). In addition to the difficulty associated with its early detection, ovarian cancer develops drug resistance easily, especially to platinum- and taxane-based drugs [4], and this disease may relapse 2–10 years after treatment. Despite the advances in multidisciplinary treatment, a significant number of patients eventually develop metastatic or recurrent diseases and the overall mortality of ovarian cancer has remained steady over the last 30 years [5]. The standard treatment for advanced epithelial ovarian cancer (EOC) is based on maximum debulking surgery, followed by platinum and taxane-based chemotherapy [6]. Chemoresistance frequently occurs in recurrent patients, and the mechanism underlying the chemoresistance of ovarian cancer has not yet been elucidated [7]. The emergence of chemoresistance to anticancer drugs is an important factor contributing to the poor survival rates of EOC. Therefore, chemoresistance is a major obstacle in ovarian cancer therapy, and overcoming chemoresistance is an important goal in ovarian cancer therapy [8].

Focal adhesions are large and dynamic plasma membrane-associated macromolecular assemblies through which integrins and scaffold proteins link the actin cytoskeleton to the extracellular matrix. The coordinated and dynamic regulation of focal adhesions is required for cell migration, which plays an essential role in cancer metastasis [9, 10]. Metastasis is intricately linked with resistance to chemotherapy, both clinically and biologically, but the molecular basis for this link is unknown [11–13]. Numerous studies have demonstrated that acquired chemoresistance is associated with the metastatic and migratory phenotypes of in human colon adenocarcinoma and ovarian cancer cell lines [14, 15]. In our previous study, we demonstrated that chemoresistant ovarian cancer cells acquired epithelial-to-mesenchymal transition (EMT) and stemness phenotype. The chemoresistant cells exhibited an elongated mesenchymal-like morphology, fewer cell–cell junctions and high invasibility [16]. Taxol-resistant ovarian cancer cell lines have shown reduction in focal adhesion size but increase in focal adhesion kinase (FAK), microtubule dynamics, and cell attachment rate [17]. In addition, integrin adhesion molecules and focal adhesion proteins were found to be significantly upregulated in cisplatin-treated lung cancer cells [18, 19]. Evidence suggests that chemoresistant cancer cells alter their behaviors toward a more aggressive phenotype by modulating of focal adhesion dynamics.

Ca^2+^ signaling has been known to be critical in regulating focal adhesion dynamics [20]. Focal adhesion turnover is crucial in a migratory cell and is mediated by Ca^2+^-dependent assembly and disassembly [21]. Thus, the regulation of spatial and temporal characteristics of Ca^2+^ signaling is important for cell migration [22, 23]. Store-operated Ca^2+^ entry (SOCE) is the major form of extracellular Ca^2+^ influx following the depletion of endoplasmic reticulum (ER) Ca^2+^ stores in non-excitable cells to refill intracellular Ca^2+^ stores, regulate basal Ca^2+^, and execute a wide range of Ca^2+^-associated specialized activities [24, 25]. The ER Ca^2+^ sensor stromal interaction molecule 1 (STIM1), two plasma membrane Ca^2+^ channels calcium release-activated calcium modulator 1 (also known as Orai1), and transient receptor potential canonical 1 (TRPC1), are the major components involved in SOCE [26, 27]. Activated SOCE promotes cancer cell proliferation, chemoresistance, and migration [28, 29]. Recent studies have shown that SOCE is highly activated in various cancers and is associated with different cancer development and progression [30–32]. In addition, SOCE is required for chemoresistance in 5-FU or cisplatin-treated pancreatic, liver, lung, and ovarian cancer cells, suggesting that a SOCE blocker could be useful in combination with chemotherapies to treat refractory tumors [33–36]. However, the effects of SOCE contributing to chemoresistance have been rarely reported. Thus, the potential regulatory mechanism of SOCE in chemoresistance is unknown.

In this study, we examined the effects of SOCE on focal adhesion dynamics and migration in chemoresistant ovarian cancer cells. We found that focal adhesion assembly and disassembly rates, cell adhesion, and cell migration are higher in SOCE-upregulated chemoresistant ovarian cancer cells. Inhibition of SOCE attenuated focal adhesion dynamics and cell migration. Interestingly, SOCE inhibitors sensitized the resistant ovarian cancer cells to chemotherapeutic drugs.

## Materials and methods

### Cells and cell culture

Human ovarian carcinoma cell line IGROV1 was maintained in RPMI 1640 medium (GIBCO, Big Cabin, OK) supplemented with 10% fetal bovine serum (FBS; GIBCO, Big Cabin, OK), penicillin (100 IU/ml) and streptomycin (100 μg/ml) under 5% CO_2_ at 37 °C. Chemoresistant sublines (IGROV1-CP and IGROV1-SRT) were obtained by exposing IGROV1 cells to stepwise increases in cisplatin (Sigma-Aldrich, Saint Louis, MO) or SR-T100 (G&E Herbal Biotechnology, Tainan, Taiwan) concentrations. Cisplatin or SR-T100 dose was doubled after the completion of the initial concentration during a 3–6 week period, and the procedure was repeated until drug levels with significant cell death were achieved.

### DNA transfection and reagents

For transient transfection, the EGFP-tagged FAK, paxillin, vinculin, and talin plasmids were transfected into IGROV1 cells using Lipofectamine 3000 (Invitrogen, San Diego, CA) for 48 h. Thapsigargin, YM-58483, PD105606, PD151746, ALLN, and cisplatin were purchased from Sigma–Aldrich (Saint Louis, MO). Calpeptin and calpastatin were purchased from Cayman Chemical (Ann Arbor, MI). 2-APB, SKF-96365, and fura-2/AM were purchased from Invitrogen (San Diego, CA). SR-T100 was kindly provided by G&E Herbal Biotechnology (Tainan, Taiwan).

### Cell adhesion assay

Cells were starved for 12 h and then detached using 20-mM EDTA for 30 min. Subsequently, 7.5 × 10^4^ cells were seeded in a poly-L-lysine coated 3-cm dish in RPMI medium with 0.1% BSA and incubated at 37 °C for 30 min. The cells were fixed with 4% paraformaldehyde for 10 min and stained with DNA-binding fluorescent probe Hoechst 33342 for 30 min. Images of fluorescent dye-stained cell nuclei were captured using an inverted-fluorescence microscope. Adherent cells were counted in 15 randomly selected fields, and the number of cells per field was recorded. Each assay was performed from three independent experiments and analyzed as the cell number per field using the ImageJ software.

### Focal adhesion dynamics

EGFP-tagged DNA plasmids (FAK, paxillin, vinculin, talin) were transient transfected into IGROV1 cells by Lipofectamine™ 3000 reagent (Invitrogen, San Diego, CA). Live cell time-lapse focal adhesion dynamics were measured using cells with gene expression 40 h post-transfection under a TIRF microscope at 30-s intervals per image for 1 h. Finally, analysis was performed using an online open-source software, i.e., Focal Adhesion Analysis Server [37, 38]. Assembly and disassembly rates of focal adhesions were analyzed by uploading grayscale split-sequences videos with parameters such as imaging frequency, detection threshold, and minimum and maximum adhesion sizes. Data with coefficient of determination (denoted by R^2^) above 0.7 were used in this study.

### Wound healing assay

Culture inserts (ibidi, Martinsried, Germany) were applied to assess IGROV1 cell migration. The insert consisted of two wells separated by a 500-μm-thick silicon wall. IGROV1 cells were seeded at an equal density (3 × 10^4^ cells in 100 μl) with 10% FBS medium and incubated at 37 °C with 5% CO_2_ overnight. The insert was removed after the cells were well attached and formed a monolayer. The cells were then incubated in DMEM containing 10% FBS. Cell migrating into the gap (initially ~ 500 μm) was recorded every 12 h via phase-contrast microscopy. The data were collected from three independent experiments and analyzed as wound closure (%) using the ImageJ software.

### Transwell migration assay

The transwell chambers used for the migration assays contained polycarbonate filters of 8-μm pore size (BD Biosciences, San Jose, CA). A medium containing 10% FBS was placed in the lower chambers to serve as a chemoattractant. Cells (2 × 10^4^ in 500 μl serum-free medium) were placed in the upper chamber and incubated at 37 °C for 8 h. The cells that penetrated the filter were counted in 15 randomly selected fields, and the mean number of cells per field was recorded. Each assay was performed on duplicate filters, and each experiment was repeated twice.

### Intracellular Ca^2+^ measurement of store-operated Ca^2+^ entry

Cytosolic Ca^2+^ was measured at 37 °C using the fura-2 fluorescence ratio method on a single-cell fluorimeter. Cells were loaded with 2 μM fura-2/AM in DMEM culture medium at 37 °C for 30 min. ER Ca^2+^ was depleted by the addition of thapsigargin (2 μM) for 10 min in Ca^2+^-free buffer. Thereafter, Ca^2+^ influx by SOCE was triggered by an exchange with extracellular Ca^2+^ buffers (0 to 2 mM) for 5 min at time point “30 s”. The excitation wavelength was alternated between 340 nm and 380 nm using the Polychrome IV monochromator (Till Photonics, Grafelfing, Germany). The fluorescence intensity was monitored at 510 nm, stored digitally, and analyzed using the program TILLvisION 4.0 (Till Photonics, Grafelfing, Germany).

### Western blotting

Cell lysates were harvested in RIPA buffer (150 mM NaCl, 1 mM EGTA, 50 mM Tris at pH 7.4, 10% glycerol, 1% Triton X-100, 1% sodium deoxycholate, 0.1% SDS, and Complete^TM^), and subsequently analyzed by Western blotting using antibodies against STIM1, FAK, paxillin, E-cadherin, ZO-1, fibronectin (BD Biosciences, San Jose, CA), Orai1, Orai3 (Prosci, Poway, CA), Orai2, phospho-Tyr397-FAK (Enzo, Farmingdale, NY), TRPC1 (Proteintec, Rosemont, IL), vinculin, vimentin, N-cadherin (Santa Cruz, Santa Cruz, CA), talin (abcam, Cambridge, UK), phospho-Tyr18-paxillin (Invitrogen, San Diego, CA), STIM2 (Cell Signaling, Danvers, MA), and β-actin (Sigma-Aldrich, Saint Louis, MO). The immunocomplexes were then detected with horseradish peroxidase-conjugated IgG (Jackson ImmunoResearch Laboratories, West Grove, PA), and the reaction was developed using an ECL detection kit (Amersham, Piscataway, NJ) under an ImageQuant LAS 4000 system (GE Healthcare Life Sciences, Pittsburgh, PA).

### Immunofluorescence staining and TIRF microscopy

Cells were fixed with 4% buffered paraformaldehyde, and permeabilized using 0.5% Triton X-100 for 15 min. The fixed cells were blocked with CAS-Block (Invitrogen, San Diego, CA) at 25 °C for 1 h. The cells were then incubated with primary anti-FAK, anti-vinculin, anti-Orai1 (Santa Cruz, Santa Cruz, CA), anti-STIM1 (abcam, Cambridge, UK), anti-paxillin (BD Biosciences, San Jose, CA), or anti-talin (Millipore, Billerica, MA) antibody overnight at 4 °C. In addition, the cells were stained with goat anti-mouse IgG conjugated with Alexa 488 or goat anti-rabbit IgG conjugated with Alexa 594 (Molecular Probes, Eugene, OR) for 1 h. The fluorescence images of the focal adhesions were acquired and analyzed using a total internal reflection fluorescence microscope (cell^TIRF; Olympus, Tokyo, Japan) with 491 nm laser. The FV10-ASW software was used to analyze focal adhesion proteins.

### Statistical analysis

All data were reported as mean ± SEM (standard error of the mean). For statistical analysis, the Student’s *t*-test or one-way ANOVA with Dunnett’s post-hoc test was used to assess the significance of differences between groups. A *p* value < 0.05 was considered statistically significant.

## Results

### Chemoresistant IGROV1 sublines exhibit mesenchymal morphology and high migratory ability

**Fig. 1:**
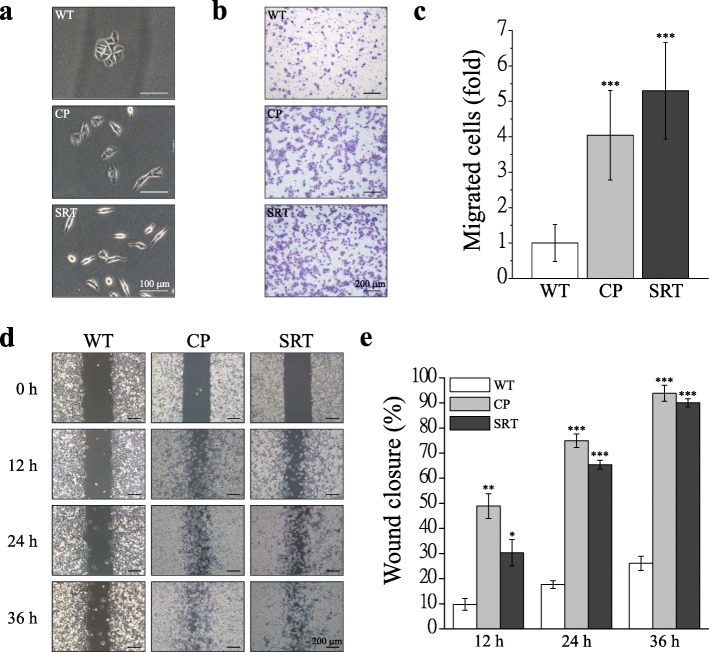
Chemoresistant IGROV1 sublines exhibit high migratory ability. IGROV1 cells (WT) resistant to 2 μM cisplatin (CP), and 2 μg/ml SR-T100 (SRT) were isolated. **a** Phase contrast images of parental and chemoresistant cells. Scale bars, 100 μm. **b** In vitro transwell migration assay. Representative photomicrographs of cells that penetrated a filter of pore size of 8 μm. Scale bars, 200 μm. **c** Migrated cells were counted in 15 random fields on the lower surface of the filters and expressed as ratio (fold) of migrated cells compared with WT. **d** Cells were seeded into silicon inserts with 10% FBS medium. Following cell adhesion, inserts were removed and incubated for 36 h. Phase images were captured every 12 h and wound spaces were analyzed using ImageJ. **e** Cellular migratory ability is presented as the percentage of wound closure. Each bar represents mean ± SEM from three independent experiments. *: significant difference between chemoresistant (CP, SRT) and parental (WT) cells. *: *p* < 0.05; **: *p* < 0.01; ***: *p* < 0.001 by Student’s *t*-test

Platinum-based chemotherapeutics is the routine treatment of ovarian cancer patients [39], and patients developing cisplatin resistance is a major clinical obstacle that cause a relapse after initial favorable responses. Cisplatin treatment induces intrastrand and interstrand DNA adducts [40], resulting in the accumulation of DNA strand breaks and ultimately cell death upon failure to activate or execute appropriate DNA repair [41]. SR-T100, a newly patented product extracted from *Solanum incanum*, which contains solamargine alkaloid as the main active ingredient, is a potent inducer of apoptosis in different cancer cells that upregulates the expression of death receptor signaling cascades [42, 43]; it downregulated Bcl-X_L_ but upregulated Bax and caused caspase-3 activation of the mitochondrial pathway [44, 45]. SR-T100 has been used as an anticancer drug for clinical therapy [46, 47]. To elucidate the underlying mechanisms of chemoresistance affecting cell migration in ovarian cancer, several chemoresistant human ovarian cancer IGROV1 sublines to cisplatin or SR-T100 were established and applied in this study. Previously, we have demonstrated chemoresistance induced EMT in ovarian cancer cells (Additional file 1: Fig. S1) [16]. In the present study, cells with chemoresistance to cisplatin and SR-T100 exhibited morphological changes, including elongated spindle-shaped morphology and diminished cell–cell junctions between cells compared to the parental IGROV1 cells (Fig. 1a). In vitro assays indicated the higher migration ability of chemoresistant IGROV1 cells in both single-cell (Fig. 1b, c) and collective cell (Fig. 1d, e) migration by transwell migration and wound healing migration assays, respectively. This indicates that the cells achieved the EMT phenotype and migratory ability during drug selection.

### Chemoresistant IGROV1 sublines change characteristics of focal adhesion molecules and exhibit high adhesive ability

**Fig. 2:**
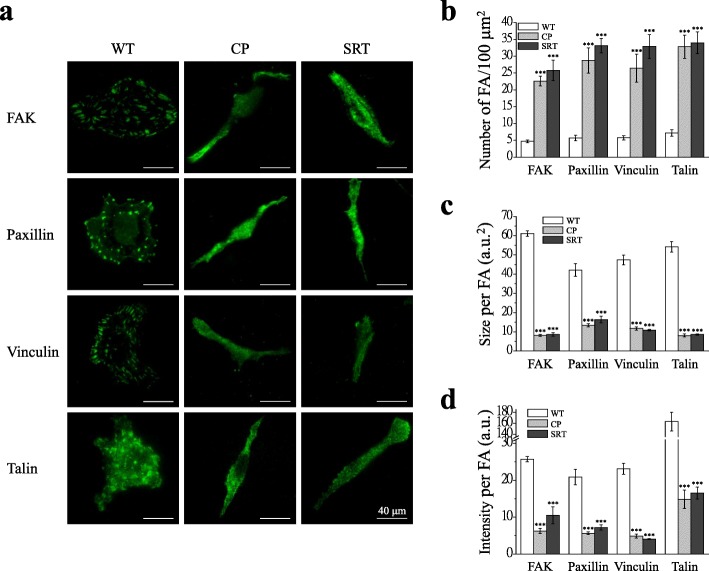
Characters of focal adhesion molecules in chemoresistant IGROV1 sublines. Immunofluorescence staining of FAK, paxillin, vinculin and talin focal adhesion molecules was performed after fixation of IGROV1 parental (WT) and chemoresistant (CP, SRT) cells. **a** Representative fluorescence images of IGROV1 cells overexpressing EGFP-tagged focal adhesion molecules (FAK, paxillin, vinculin, talin) captured under a total internal reflection fluorescence microscope (TIRFM). Scale bars, 40 μm. **b-d** Quantitative analysis of the (**b**) number, (**c**) size, and (**d**) intensity of focal adhesions. Each bar represents mean ± SEM from at least 20 cells. a.u., arbitrary unit. *: significant difference between chemoresistant (CP, SRT) and parental (WT) cells. ***: *p* < 0.001 by Student’s *t*-test

FAK, paxillin, vinculin, and talin are major components within the focal adhesion complex. The construction, organization, and coordinated and dynamic regulation of focal adhesion are required for cell migration. We aimed to clarify the effect of chemoresistance on the function of focal adhesion molecules. A total internal reflection fluorescence microscope (TIRFM), which is used for visualizing the localization or interaction of fluorescent molecules in a near-membrane region (~ 200 nm), was used to observe focal adhesion molecules. As shown by the images obtained with a TIRFM (Fig. 2a), the number of focal adhesions increased significantly in the chemoresistant cells (Fig. 2b). By contrast, the size and individual molecular intensity of the focal adhesions decreased in these chemoresistant cells (Fig. 2c, d). In addition, the chemoresistant cells exhibited strong adhesive ability compared with the parental IGROV1 cells (Additional file 2: Fig. S2).

### Chemoresistant IGROV1 sublines enhance dynamics of focal adhesions

**Fig. 3:**
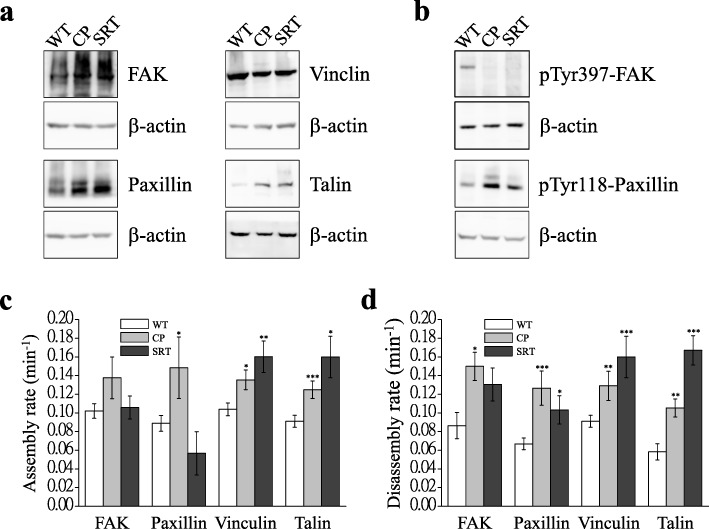
Chemoresistant IGROV1 sublines enhance dynamics of focal adhesions. **a-b** FAK, paxillin, vinculin, talin, phosphorylated FAK (pTyr397-FAK), and phosphorylated paxillin (pTyr118-Paxillin) were detected using immunoblotting in IGROV1 parental (WT) and chemoresistant (CP, SRT) cells. β-actin served as the internal control. **c-d** Real-time recordings by TIRFM was used to evaluate the turnover of EGFP-tagged focal adhesion molecules (FAK, paxillin, vinculin, talin) in IGROV1 cells. Quantitative analysis of (**c**) assembly and (**d**) disassembly rates of focal adhesion molecules in IGROV1 cells. Each bar represents mean ± SEM from at least 15 cells. *: significant difference between chemoresistant (CP, SRT) and parental (WT) cells. *: *p* < 0.05; **: *p* < 0.01; ***: *p* < 0.001 by Student’s *t*-test

Western blotting showed the upregulation of focal adhesion molecules (FAK, paxillin, and talin) in chemoresistant IGROV1 cells rather than in the parental cells (Fig. 3a). Meanwhile, phosphorylation of paxillin (pTyr118-Paxillin) and dephosphorylation of FAK (pTyr397-FAK) were observed in chemoresistant IGROV1 cells (Fig. 3b). The dynamic regulation of focal adhesions represents a critical step in the decision process regarding cell migration. In this study, we investigated focal adhesion turnover in living cells. Real-time recordings by TIRFM were performed to assess the turnover of focal adhesions with EGFP-tagged focal adhesion molecules. We found that chemoresistant IGROV1 cells had higher assembly (Fig. 3c) and disassembly (Fig. 3d) rates of focal adhesions (Additional files 5, 6, 7: Videos S1-S3). Comprehensively, these results indicate that the characters and function of the focal adhesions confer more effective focal adhesion formations and highly migratory potencies in chemoresistant cells.

### SOCE is critical for regulating migration ability of ovarian cancer cells

**Fig. 4:**
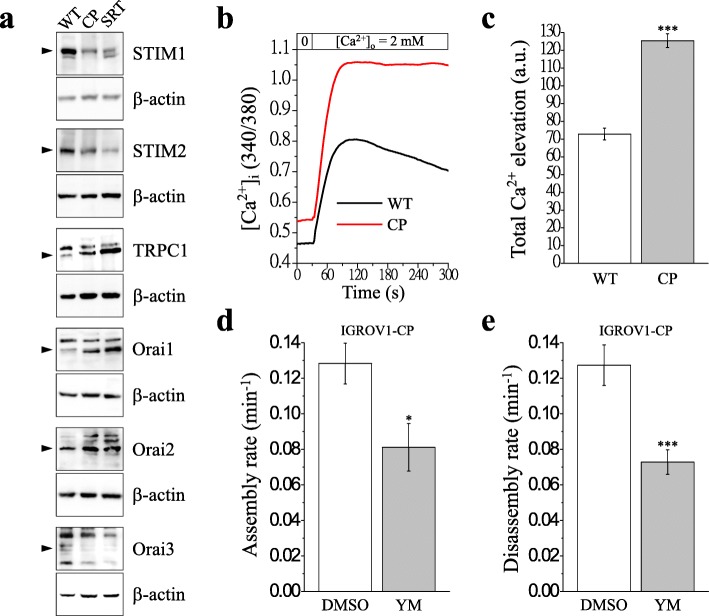
Enhancement of store-operated Ca^2+^ entry in chemoresistant IGROV1 sublines. **a** STIM1, STIM2, TRPC1, Orai1, Orai2, and Orai3 were detected using immunoblotting in IGROV1 parental (WT) and chemoresistant (CP, SRT) cells. β-actin served as the internal control. **b** Pre-incubation of IGROV1 parental (WT) and chemoresistant (CP) cells with 2 μM fura-2/AM at 37 °C for 30 min for cytosolic Ca^2+^ measurement using a single-cell fluorimeter. Depletion of ER lumen-resident Ca^2+^ was induced by treating cells in Ca^2+^-free buffer with 2 μM thapsigargin for 10 min. Representative tracings show the subsequent elevation of Ca^2+^ and indicated that SOCE occurred during the exchange of Ca^2+^-free buffer to 2 mM Ca^2+^ buffer for 5 min. The data in representative curves for the measurement of SOCE from three independent experiments (where, *n* ≥ 60 cells). **c** SOCE-mediated total Ca^2+^ elevation was calculated from area under the curve. a.u., arbitrary unit. **d-e** IGROV1-CP cells overexpressing EGFP-tagged paxillin were pretreated with 10 μM YM-58483 for 30 min. Time-lapse images were captured under a total internal reflection fluorescence microscope (TIRFM). Quantitative analysis of the (**d**) assembly and (**e**) disassembly rates of paxillin in IGROV1-CP cells. Each bar represents mean ± SEM from at least 20 cells. *: significant difference between chemoresistant (CP) and parental (WT) cells. **: *p* < 0.01; ***: *p* < 0.001 by Student’s *t*-test

SOCE has been implicated in cancer cell migration and tumor metastasis. STIMs, TRPC1, and Orais are critical in the regulation of SOCE. To understand whether SOCE contributes to higher focal adhesion dynamics and cell migration in chemoresistant cells, the expression of SOCE-related molecules was examined. Western blotting results demonstrated that the levels of STIM1, STIM2, and Orai3 decreased in chemoresistant cells. By contrast, chemoresistant cells increased the levels of TRPC1, Orai1, and Orai2 expression (Fig. 4a). Single-cell Ca^2+^ imaging for SOCE was applied to examine the difference between chemoresistant cells and parental IGROV1 cells (Fig. 4b). The results indicate that SOCE-mediated Ca^2+^ influx following thapsigargin-mediated ER Ca^2+^ store depletion in IGROV1-CP cells is 1.7-fold higher than that in IGROV1-WT cells (Fig. 4c).

**Fig. 5:**
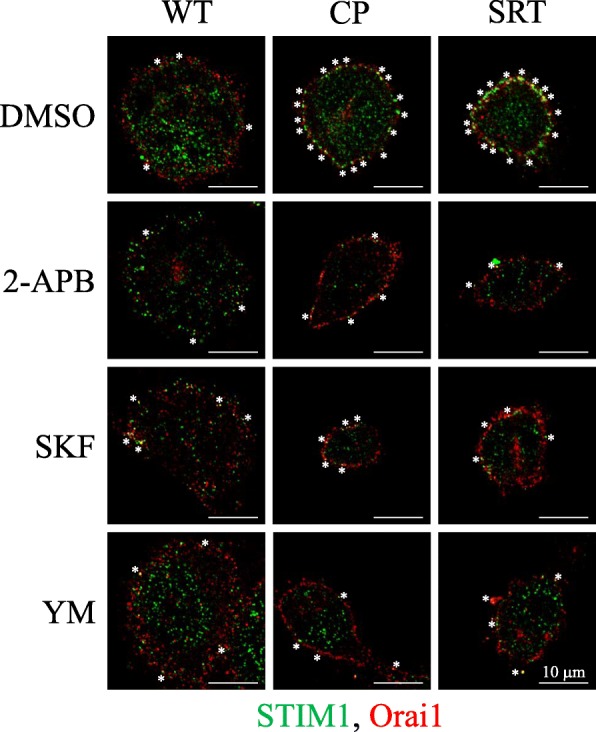
SOCE inhibitors reduced interaction between STIM1 and Orai1 in IGROV1 cells. Pretreatment of SOCE inhibitors (2 μΜ SKF-96365, 10 μΜ YM-58483, 0.1 μM 2-APB) on parental (WT) and chemoresistant (CP, SRT) IGROV1 cells for 1 h. Afterwards, cells were treated with 2 μM thapsigargin for 5 min, and then fixed with 4% paraformaldehyde. Immunofluorescence staining was performed to label STIM1 and Orai1, and the fluorescence images were obtained using confocal microscopy. Scale bars, 10 μm

SOCE inhibitors, i.e., SKF-96365, YM-58483, and 2-APB, were used in this study to verify the effects of SOCE on focal adhesion dynamics and cell migration. In vitro cell migration assays, especially wound healing assays, may be facilitated by cell proliferation and cell migration. To avoid side effects from cell proliferation in a migration assay, relatively low and sublethal doses (2 μΜ SKF-96365, 10 μΜ YM-58483, and 0.1 μM 2-APB) of these SOCE inhibitors were applied and did not affect cell proliferation for 3–4 days (data not shown). We found that these SOCE inhibitors could downregulate the SOCE-mediated Ca^2+^ influx up to 94% (SKF-96365: 48%; YM-58483: 94%; 2-APB: 67%) in IGROV1-WT cells (Additional file 3: Fig. S3). The results of immunofluorescence staining also showed ER-plasma membrane translocation of STIM1 protein and its interaction with Orai1 channel in response to thapsigargin-induced depletion of ER Ca^2+^ stores in chemoresistant IGROV1 sublines (IGROV1-CP and IGROV1-SRT) is higher than that in IGROV1-WT cells. These alterations can be blocked in the presence of SOCE inhibitor (SKF-96365, YM-58483, and 2-APB). In addition, SOCE inhibitors also reduced the formation of STIM1 puncta after thapsigargin treatment (Fig. 5). Furthermore, in vitro migration assays exhibited the blocking of transwell migration (Additional file 4: Fig. S4a, b) and wound healing migration (Additional file 4: Fig. S4c, d) by SOCE inhibitors in IGROV1-WT cells. This indicates that SOCE is critical in regulating cell migration.

### Inhibition of SOCE decrease migration ability of chemoresistant IGROV1 cell

**Fig. 6:**
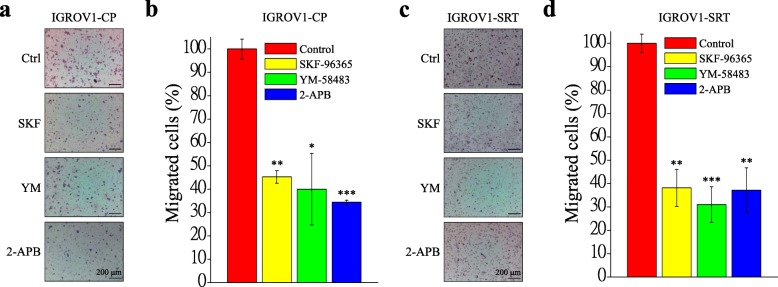
SOCE inhibitors decrease migration ability of chemoresistant IGROV1 cells in transwell assay. In vitro transwell migration assay was performed to evaluate the effect of SOCE inhibitors (2 μΜ SKF-96365, 10 μΜ YM-58483, 0.1 μM 2-APB) on chemoresistant IGROV1 (CP, SRT) cells. **a, c** Representative photomicrographs of (**a**) IGROV1-CP and (**c**) IGROV1-SRT cells that penetrated a filter of pore size of 8 μm. Scale bars, 200 μm. **b, d** Migrated (**b**) IGROV1-CP and (**d**) IGROV1-SRT cells were counted in 15 random fields on the lower surface of the filters and expressed as percentage (%) of SOCE inhibitor pretreated cells compared with DMSO control (Ctrl). Each bar represents mean ± SEM from three independent experiments. *: significant difference between cells treated with SOCE inhibitors and DMSO control (Ctrl). *: *p* < 0.05; **: *p* < 0.01; ***: *p* < 0.001 by Student’s *t*-test

**Fig. 7:**
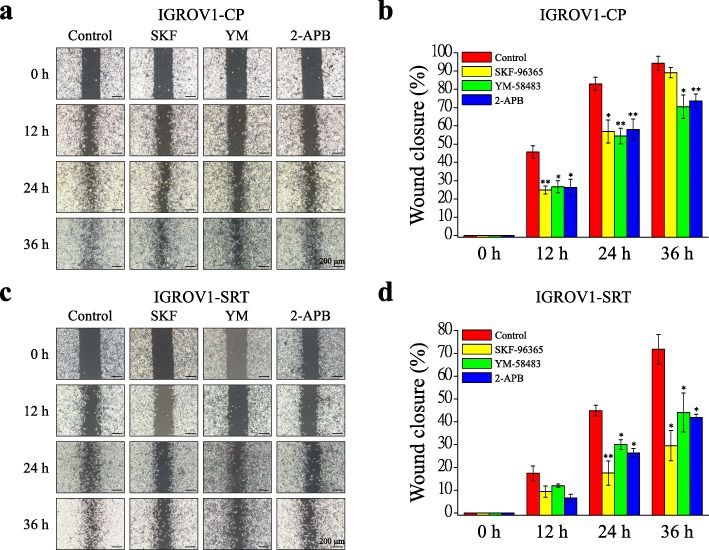
SOCE inhibitors decrease migration ability of chemoresistant IGROV1 cells in wound healing assay. In vitro wound healing migration assay was performed to evaluate the effect of SOCE inhibitors (2 μΜ SKF-96365, 10 μΜ YM-58483, 0.1 μM 2-APB) on chemoresistant IGROV1 (CP, SRT) cells. **a, c** (**a**) IGROV1-CP and (**c**) IGROV1-SRT cells were seeded into silicon inserts with 10% FBS medium. Following cell adhesion, inserts were removed and incubated for 36 h. Phase images were captured every 12 h and wound spaces were analyzed using ImageJ. **b, d** Cellular migratory ability is presented as the percentages of wound closure. Each bar represents mean ± SEM from three independent experiments. *: significant difference between cells treated with SOCE inhibitors and DMSO control (Control). *: *p* < 0.05; **: *p* < 0.01 by Student’s *t*-test

**Fig. 8:**
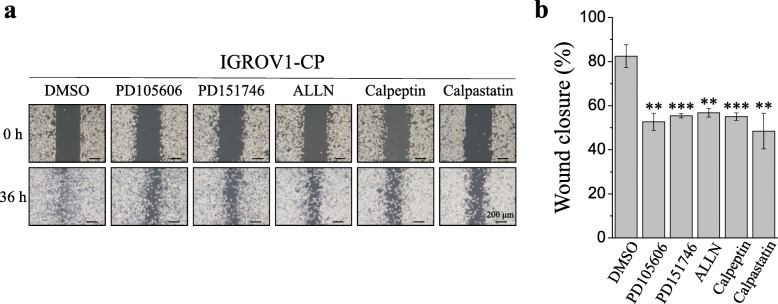
Calpain inhibitors reduce cell migration in chemoresistant IGROV1 cells. The calpain inhibitors were applied to examine their effects on cell migration by wound healing assay. Chemoresistant IGROV1 cells (CP, SRT) were seeded into silicon inserts with 10% FBS medium. Following cell adhesion, cells under pretreatment with calpain inhibitors for 30 min, including 50 μM PD105606, 50 μM PD151746, 10 μM ALLN, 10 μM calpeptin, and 50 μM calpastatin. Non-calpain inhibitor pretreated cells were treated with DMSO. Then, inserts were removed and incubated cells in medium for 36 h. **a** Phase images were captured at 36 h and wound spaces were analyzed using ImageJ. **b** Cellular migratory ability is presented as the percentage of wound closure. Each bar represents mean ± SEM from three independent experiments. *: significant difference between calpain inhibitor pretreated cells and non-calpain inhibitor pretreated cells. **: p < 0.01; ***: p < 0.001 by Student’s *t*-test

**Fig. 9:**
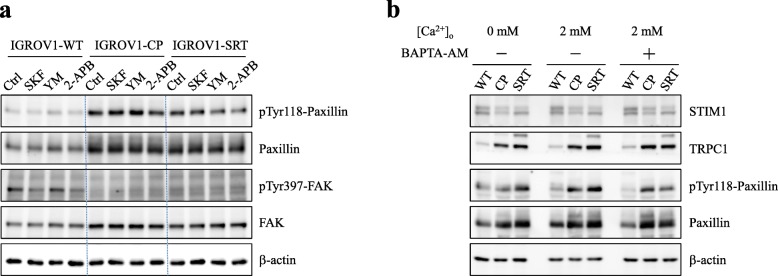
Ca^2+^ signaling does not affect the activity of focal adhesion molecules and the expression of SOCE components. **a** Pretreatment of SOCE inhibitors (2 μΜ SKF-96365, 10 μΜ YM-58483, 0.1 μM 2-APB) on IGROV1 parental (WT) and chemoresistant (CP, SRT) IGROV1 cells for 24 h. FAK, paxillin, phosphorylated FAK (pTyr397-FAK), and phosphorylated paxillin (pTyr118-Paxillin) were detected using immunoblotting. β-actin served as the internal control. **b** The intracellular Ca^2+^ chelator, BAPTA-AM (1 μM), was used to deplete intracellular Ca^2+^. IGROV1 cells were cultured in medium with (2 mM) or without (0 mM) Ca^2+^ for 48 h. After collection of protein lysates, STIM1, TRPC1, pTyr118-Paxinllin and paxillin were detected using immunoblotting in IGROV1 parental (WT) and chemoresistant (CP, SRT) cells. β-actin served as the internal control

YM-58483 is the most efficient SOCE inhibitor to inhibit SOCE and cell migration in IGROV1-WT cells (Additional files 3, 4: Figs. S3, S4). In this study, we used the SOCE inhibitor YM-58483 to investigate focal adhesion turnover and cell migration in chemoresistant IGROV1-CP cells. Real-time TIRFM imaging showed that YM-58483 downregulated the assembly (Fig. 4d, Additional file 8: Video S4) and disassembly (Fig. 4e, Additional file 9: Video S5) rates of focal adhesions. Moreover, all of these SOCE inhibitors could inhibit transwell migration (Fig. 6) and wound healing migration (Fig. 7) in both IGROV1-CP and IGROV1-SRT chemoresistant cells. Five calpain inhibitors were used to examine the critical role of calpain in regulation of cell migration. All calpain inhibitors significantly inhibited migration in wound healing of the chemoresistant IGROV1-CP cells. Among them, the inhibitory effects on calpain mediated cell migration from high to low is calpastatin (41%), PD105606 (37%), calpeptin (34%), PD151746 (33%), and ALLN (31%), respectively (Fig. 8). However, SOCE inhibitors did not affect the phosphorylation and expression of focal adhesion molecules (Fig. 9a). Moreover, the use of extracellular Ca^2+^-free medium or intracellular Ca^2+^ chelator BAPTA-AM did not affect the expression level of SOCE components (STIM1, TRPC1) and focal adhesion molecules (pTyr118-Paxillin, paxillin) (Fig. 9b).

### Inhibition of SOCE sensitize chemoresistant IGROV1 cells to chemotherapeutic agents

**Fig. 10:**
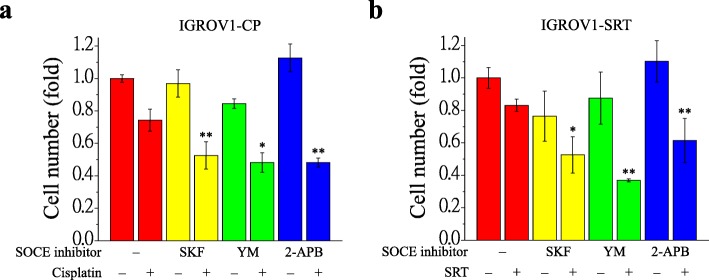
Inhibition of SOCE sensitize chemoresistant IGROV1 cells to chemotherapeutic agents. Cell number was counted to evaluate the effect of SOCE inhibitors on chemotherapeutic agents-mediated cytotoxicity in chemoresistant IGROV1 (CP, SRT) cells. Combination treatment of SOCE inhibitors (2 μΜ SKF-96365, 10 μΜ YM-58483, 0.1 μM 2-APB) and chemotherapeutic agents (2 μΜ cisplatin, 15 μg/ml SR-T100) for 48 h in (**a**) IGROV1-CP and (**b**) IGROV1-SRT cells. Nuclear staining by DAPI showed existing cells and cell number was quantitative analyzed using ImageJ. Each bar represents mean ± SEM from three independent experiments. *: significant difference between cells treated with or without SOCE inhibitors (SKF-96365, YM-58483, 2-APB). *: *p* < 0.05; **: *p* < 0.01 by Student’s *t*-test

Cancer metastasis is the primary cause of morbidity and mortality, whereas chemoresistance is a major obstacle in ovarian cancer therapy. Our findings suggest that SOCE inhibitors could inhibit focal adhesion dynamics and cell migration significantly. We asked whether inhibiting SOCE could be an effective therapeutic strategy in chemoresistant ovarian cancer. Sublethal doses of various SOCE inhibitors were applied for combinational therapy. As shown in Fig. 10, all SOCE inhibitor combinations with chemotherapeutic agents (cisplatin and SR-T100) decreased the cell number in both IGROV1-CP (Fig. 10a) and IGROV1-SRT (Fig. 10b) chemoresistant cells.

## Discussion

Our studies demonstrated the relationship between chemoresistance and migration ability in terms of focal adhesion dynamics and SOCE. We first confirmed that the spindle-like chemoresistant cells demonstrated have better migration ability (Fig. 1), and cells with chemoresistance to cisplatin and SR-T100 exhibited an EMT phenotype (Additional file 1: Fig. S1). The presence of small and highly transient focal adhesions is a marker of highly migratory cells. Furthermore, we demonstrated that the focal adhesions of chemoresistant cells exhibit the following properties: higher density, smaller size, and strong adhesive force (Fig. 2, Additional file 2: Fig. S2), which are characters of highly migratory cells [48, 49]. These results imply that these chemoresistant cells have more rapid focal adhesions dynamics. Indeed, we demonstrated that this type of cellular focal adhesion can change more effectively and rapidly according to assembly and disassembly rates (Fig. 3c, d). In addition, we observed the upregulation of FAK, paxillin and talin, and the phosphorylation/activation of paxillin (pTyr118-Paxillin) in chemoresistant cells (Fig. 3a, b). Paxillin phosphorylation is critical in determining a cell’s ability to migrate and hence has been linked to processes such as wound repair and tumor metastasis [50, 51]. By contrast, the dephosphorylation of FAK at 397 (Tyr397-FAK) was indicated in chemoresistant cells although many studies have implicated FAK as a positive regulator of tumor cell migration and invasion by the phosphorylation of FAK at 397 (pTyr397-FAK) [52, 53]. However, new insights suggest that FAK inhibits cell migration and invasion [54, 55]. FAK can negatively regulate cancer cell migration under certain oncogenic signaling by the dephosphorylation of FAK at Y397 and facilitation of focal adhesion turnover at the leading edge of cells [56–58]. The evidence above supports the high migratory ability of chemoresistant cells in this study.

Ca^2+^ signaling is critical in regulating focal adhesion dynamics [21], and SOCE upregulation results in cancer cell migration, invasion, and metastasis [59]. In this study, we demonstrated not only the upregulation of SOCE but also that of SOCE-related SOC channels, such as TRPC1, Orai1, and Orai2 (Fig. 4a–c). We have demonstrated that SOCE inhibitors (SKF-96365, YM-58483, and 2-APB) reduced interaction between STIM1 and Orai1 in IGROV1 cells (Fig. 5). Subsequently, we used SOCE inhibitors to treat chemoresistant cells and found decreased migration abilities. All SOCE inhibitors (SKF-96365, YM-58483, and 2-APB) could decrease SOCE-mediated Ca^2+^ influx (Additional file 3: Fig. S3) and block the migration ability (Additional file 4: Fig. S4) of chemosensitive ovarian cancer cells (IGROV1-WT). Among them, YM-58483 demonstrated the best inhibitory effect of SOCE (Additional file 3: Fig. S3), which is also reflected in the inhibition of cell migration (Additional file 4: Fig. S4). The present data emphasize the importance of SOCE in regulating cancer cell migration. Furthermore, our data showed that all SOCE inhibitors decreased focal adhesion dynamics (Fig. 4d, e) and cell migration significantly (Figs. 6, 7) in both chemoresistant cells (IGROV1-CP and IGROV1-SRT). In addition, we also showed that calpain mediated focal adhesion degradation is the key step for chemoresistant cell migration (Fig. 8). These data indicate that chemoresistant cells may enhance their migratory ability through SOCE upregulation-mediated calpain activation. The reduction of the intracellular Ca^2+^ concentration would not affect the expression levels of SOCE components and focal adhesion molecules in parental and chemoresistant ovarian cancer cells, which may come from the regulation of other factors in the chemoresistance process (Fig. 9). In addition to the promoting role of SOCE on cell migration, we examined the effect of SOCE of chemoresistant cells on chemotherapy response. After treating the SOCE inhibitors, we found that the chemoresistant cells were more sensitive to chemotherapeutic drugs (Fig. 10). Previous studies have identified that Orai1/STIM1 expression and SOCE are increased in ovary carcinoma cells, while Akt dependent upregulation of SOCE contributes to the therapy resistance [60]. SOCE-induced P-glycoprotein expression mediated paclitaxel chemoresistance in breast cancer cells [61]. In addition, SOCE is required for chemoresistance in 5-fluouracil or cisplatin-treated pancreatic, liver, lung, and ovarian cancer cells [33–36]. Mechanisms by which the SOCE affects chemoresistance include Ca^2+^ overload, multidrug resistance (MDR) [13, 61], autophagy, modulation of MAPK and PI3K-Akt/Sgk signaling pathways [60, 62], activation of NF-κB, c-myc, and p53 transcription factors [63–65], and EMT [13]. It suggests that a Ca^2+^ blocker could be useful in combination with chemotherapies to treat refractory tumors. Our previous studies demonstrated that SOCE plays a critical role in the formation of cell polarity during directional cell migration [28]. Focal adhesion degradation involves direct proteolysis of focal adhesion molecules by Ca^2+^-mediated calpain activation, followed by disassembly of focal adhesion molecules [21]. In this study, we found the Ca^2+^ influx caused by SCOE affects the migration of chemoresistant cells through the regulation of focal adhesion dynamics. This is an evidence to suggest that SOCE-mediated dynamics of focal adhesion molecules by calpain activation is involved in the regulation of resistant cell migration. Increasing evidences have demonstrated the effects of SOCE on several cancer hallmarks and cancer-related signaling pathways. Recent studies indicated that SOCE regulates proliferation and metastasis and also that SOCE inhibitors can potentially be applied in cancer treatment [35, 65, 66].

## Conclusion

Many studies have proposed the mechanism underlying the drug resistance of ovarian cancer [67, 68]. A new treatment for recurrent and resistant ovarian cancer and improvement in its poor prognosis are urgently required. Our studies demonstrated the participation of SOCE in the improved focal adhesion dynamics and migration and chemoresistance of chemoresistant cancer cells. In the future, researchers can potentially utilize SOCE inhibitors to render chemoresistant cells more sensitive to chemotherapeutic drugs, which will in turn contribute to the development of ovarian cancer therapies.

## Supplementary information

**Additional file 1 **: **Fig. S1.** Chemoresistant IGROV1 sublines exhibit characteristics of epithelial-to-mesenchymal transition (EMT). **a** Phase contrast images of parental (WT) and chemoresistant (CP, SRT) IGROV1 cells. Scale bars, 100 μm. **b** Epithelial markers (E-cadherin, ZO-1; blue rectangles) and mesenchymal marker (vimentin, fibronectin, and N-cadherin; red rectangles) were detected using immunoblotting in IGROV1 parental (WT) and chemoresistant (CP, SRT) cells. β-actin served as the internal control.

**Additional file 2 **: **Fig. S2.** Chemoresistant IGROV1 sublines exhibit high adhesive ability. Cell adhesion assay was performed 30 min after seeding to evaluate the adhesion ability of IGROV1 parental (WT) and chemoresistant cells (CP, SRT). **a** Nuclear staining by DAPI showed remaining cells after PBS washing. **b** Quantitative analyses of adherent cells per field. Each bar represents mean ± SEM from at least 300 cells of three independent experiments. *: significant difference between chemoresistant (CP, SRT) and parental (WT) cells. ***: *p* < 0.001 by Student’s *t*-test.

**Additional file 3 **: **Fig. S3.** Effect of SOCE inhibitors on Ca^2+^ elevation in IGROV1-WT cells. **a** Pre-incubation of IGROV1-WT cells with 2 μM fura-2/AM and SOCE inhibitors (2 μΜ SKF-96365, 10 μΜ YM-58483, 0.1 μM 2-APB) at 37 °C for 30 min for cytosolic Ca^2+^ measurement using a single-cell fluorimeter. Depletion of ER lumen-resident Ca^2+^ was induced by treating cells in Ca^2+^-free buffer with 2 μM thapsigargin for 10 min. Representative tracings show the subsequent elevation of Ca^2+^, indicating that SOCE occurred during the exchange of Ca^2+^-free buffer to 2 mM Ca^2+^ buffer for 5 min. The data in representative curves for the measurement of SOCE from three independent experiments. **b** SOCE-mediated total Ca^2+^ elevation was calculated from area under the curve. a.u., arbitrary unit. Each bar represents mean ± SEM from at least 120 cells. *: significant difference between cells treated with SOCE inhibitors and DMSO control (Ctrl). ***: *p* < 0.001 by Student’s *t*-test.

**Additional file 4 **: **Fig. S4.** SOCE inhibitors decrease migration ability of IGROV1-WT cells. In vitro (**a, b**) transwell migration assay and (**c, d**) wound healing assay were performed to evaluate the effect of SOCE inhibitors (2 μΜ SKF-96365, 10 μΜ YM-58483, 0.1 μM 2-APB) on IGROV1-WT cells. **a** Representative photomicrographs of cells that penetrated a filter of pore size 8 μm. Scale bars, 200 μm. **b** Migrated cells were counted in 15 random fields on the lower surface of the filters and expressed as a percentage (%) of SOCE inhibitor pretreated cells compared with DMSO control (Ctrl). **c** Cells were seeded into silicon inserts with 10% FBS medium. Following cell adhesion, inserts were removed and incubated for 96 h. Phase images were captured every 24 h and wound spaces were analyzed using ImageJ. **d** Cellular migratory ability is presented as the percentage of wound closure. Each bar represents mean ± SEM from three independent experiments. *: significant difference between cells treated with SOCE inhibitors and DMSO control (Ctrl). *: *p* < 0.05; **: *p* < 0.01; ***: *p* < 0.001 by Student’s *t*-test.

**Additional file 5 **: **Video S1.** Dynamics of EGFP-vinculin in IGROV1-WT cells. Time-lapse TIRFM imaging of EGFP-tagged vinculin focal adhesion molecules at 30-s intervals per frame for 1 h in IGROV1-WT cells.

**Additional file 6 **: **Video S2.** Dynamics of EGFP-vinculin in IGROV1-CP cells. Time-lapse TIRFM imaging of EGFP-tagged vinculin focal adhesion molecules at 30-s intervals per frame for 1 h in IGROV1-CP cells.

**Additional file 7 **: **Video S3.** Dynamics of EGFP-vinculin in IGROV1-SRT cells. Time-lapse TIRFM imaging of EGFP-tagged vinculin focal adhesion molecules at 30-s intervals per frame for 1 h in IGROV1-SRT cells.

**Additional file 8 **: **Video S4.** Dynamics of EGFP-vinculin in chemoresistant IGROV1-CP cells. Time-lapse TIRFM imaging of EGFP-tagged vinculin focal adhesion molecules at 30-s intervals per frame for 1 h in IGROV1-CP cells.

**Additional file 9 **: **Video S5.** SOCE inhibitor attenuates dynamics of EGFP-vinculin in chemoresistant IGROV1-CP cells. Cells were pretreated with 10 μM YM-58483, a SOCE inhibitor, for 30 min before TIRFM imaging. Time-lapse TIRFM imaging of EGFP-tagged vinculin focal adhesion molecules at 30-s intervals per frame for 1 h in IGROV1-CP cells.

## Data Availability

Not applicable.

## Abbreviations

EMT: epithelial-to-mesenchymal transition
EOC: epithelial ovarian cancer
ER: endoplasmic reticulum
FAK: focal adhesion kinase
SOCE: store-operated Ca^2+^ entry
STIM1: stromal interaction molecule 1
TIRFM: total internal reflection fluorescence microscope
TRPC1: transient receptor potential canonical 1
