# Supplementary figures and images for: Chemoresistant ovarian cancer enhances its migration abilities by increasing store-operated Ca2+ entry-mediated turnover of focal adhesions
Source: J Biomed Sci. 2020 Feb 21;27:36. doi: 10.1186/s12929-020-00630-5 (PMC7033940; doi:10.1186/s12929-020-00630-5)

**a**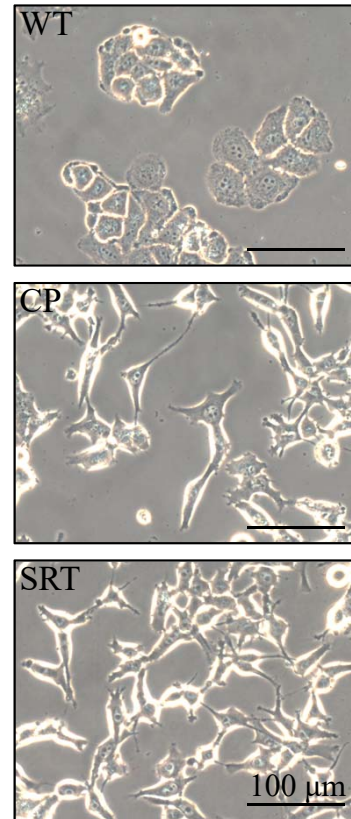**b**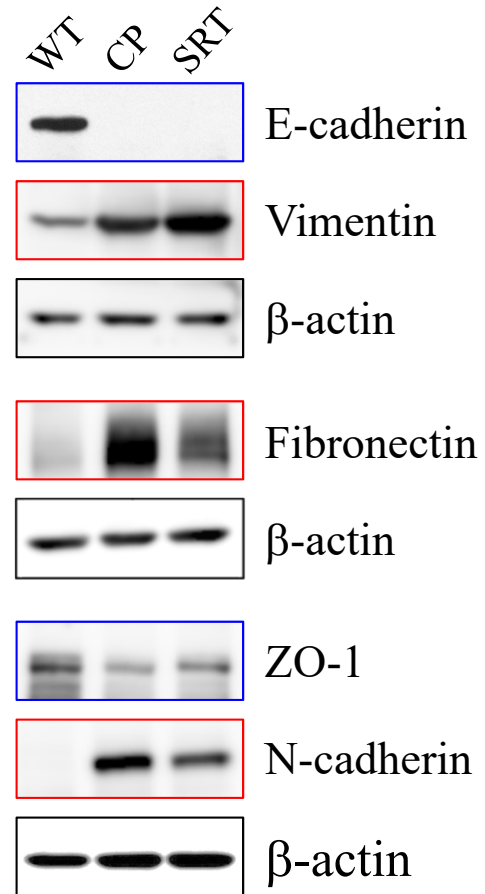

Supplement: Supplementary file 1 — Additional file 1 : Fig. S1. Chemoresistant IGROV1 sublines exhibit characteristics of epithelial-to-mesenchymal transition (EMT). a Phase contrast images of parental (WT) and chemoresistant (CP, SRT) IGROV1 cells. Scale bars, 100 μm. b Epithelial markers (E-cadherin, ZO-1; blue rectangles) and mesenchymal marker (vimentin, fibronectin, and N-cadherin; red rectangles) were detected using immunoblotting in IGROV1 parental (WT) and chemoresistant (CP, SRT) cells. β-actin served as the internal control. [file 12929_2020_630_MOESM1_ESM.pdf]

**a**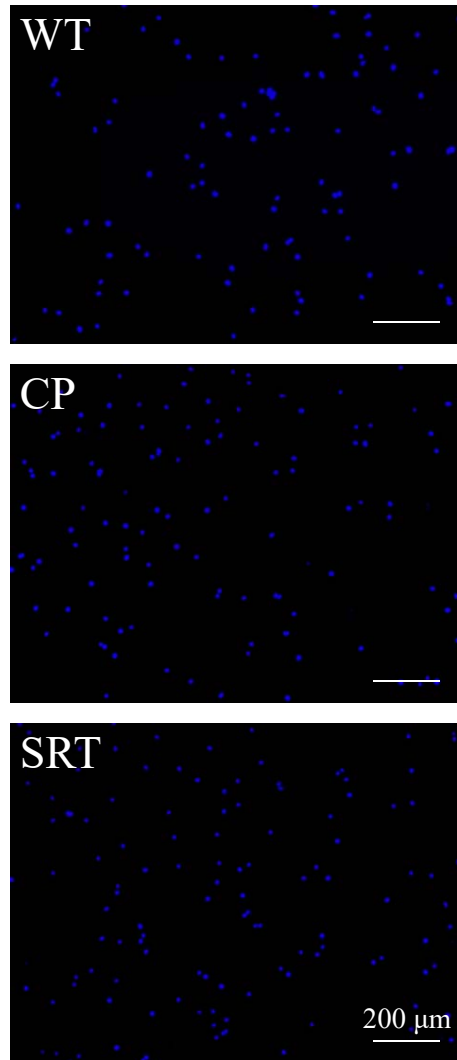**b**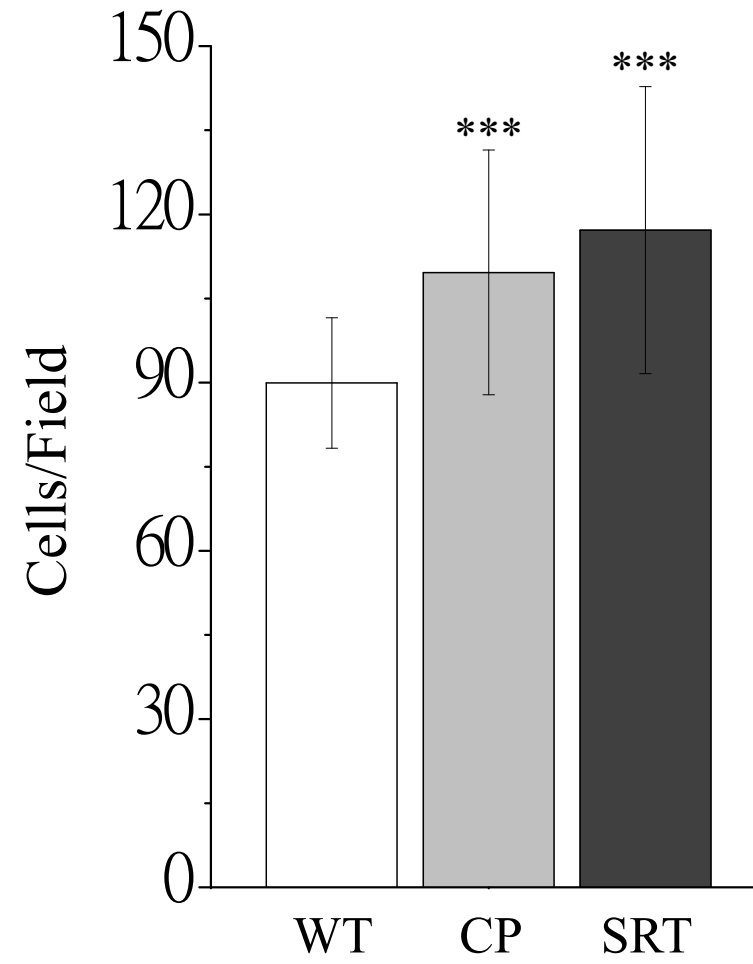

Supplement: Supplementary file 2 — Additional file 2 : Fig. S2. Chemoresistant IGROV1 sublines exhibit high adhesive ability. Cell adhesion assay was performed 30 min after seeding to evaluate the adhesion ability of IGROV1 parental (WT) and chemoresistant cells (CP, SRT). a Nuclear staining by DAPI showed remaining cells after PBS washing. b Quantitative analyses of adherent cells per field. Each bar represents mean ± SEM from at least 300 cells of three independent experiments. *: significant difference between chemoresistant (CP, SRT) and parental (WT) cells. ***: p < 0.001 by Student’s t-test. [file 12929_2020_630_MOESM2_ESM.pdf]

**a**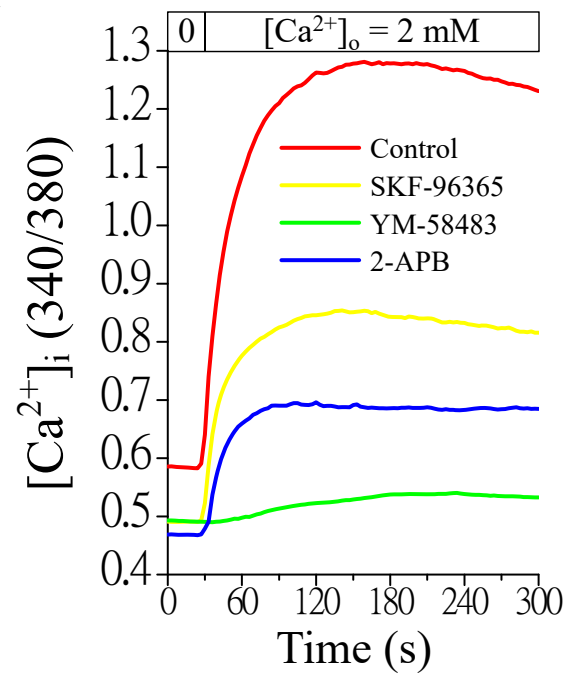**b**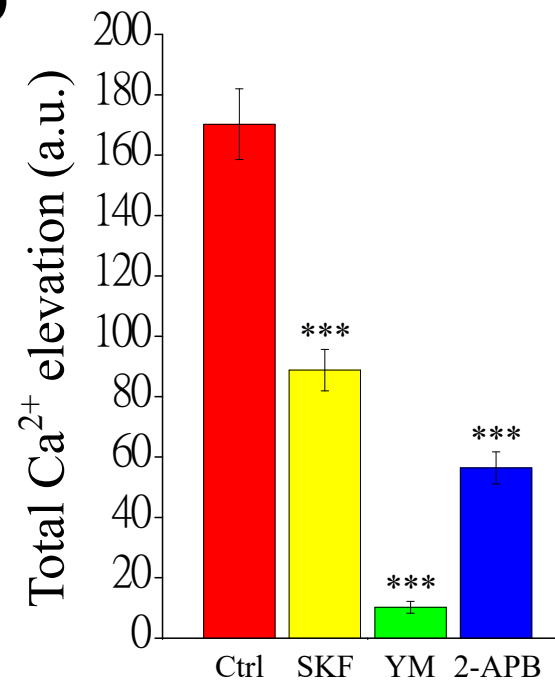

Supplement: Supplementary file 3 — Additional file 3 : Fig. S3. Effect of SOCE inhibitors on Ca2+ elevation in IGROV1-WT cells. a Pre-incubation of IGROV1-WT cells with 2 μM fura-2/AM and SOCE inhibitors (2 μΜ SKF-96365, 10 μΜ YM-58483, 0.1 μM 2-APB) at 37 °C for 30 min for cytosolic Ca2+ measurement using a single-cell fluorimeter. Depletion of ER lumen-resident Ca2+ was induced by treating cells in Ca2+-free buffer with 2 μM thapsigargin for 10 min. Representative tracings show the subsequent elevation of Ca2+, indicating that SOCE occurred during the exchange of Ca2+-free buffer to 2 mM Ca2+ buffer for 5 min. The data in representative curves for the measurement of SOCE from three independent experiments. b SOCE-mediated total Ca2+ elevation was calculated from area under the curve. a.u., arbitrary unit. Each bar represents mean ± SEM from at least 120 cells. *: significant difference between cells treated with SOCE inhibitors and DMSO control (Ctrl). ***: p < 0.001 by Student’s t-test. [file 12929_2020_630_MOESM3_ESM.pdf]

**a**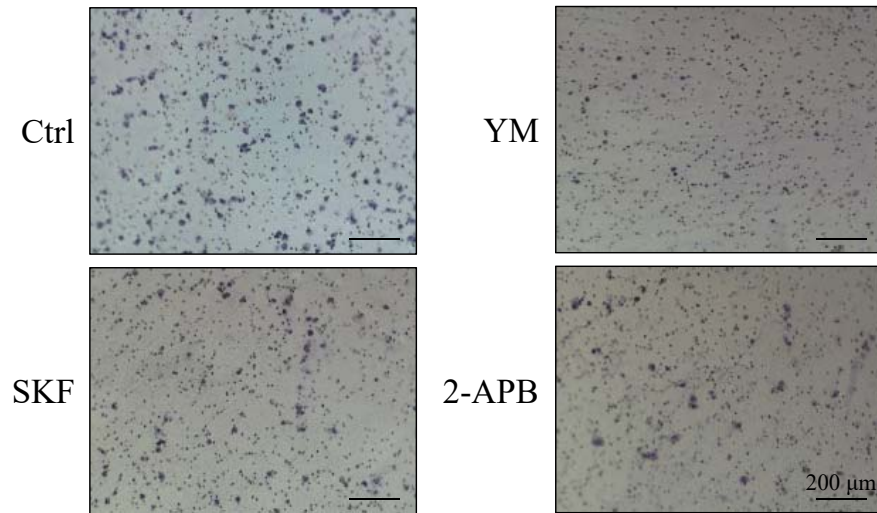**b**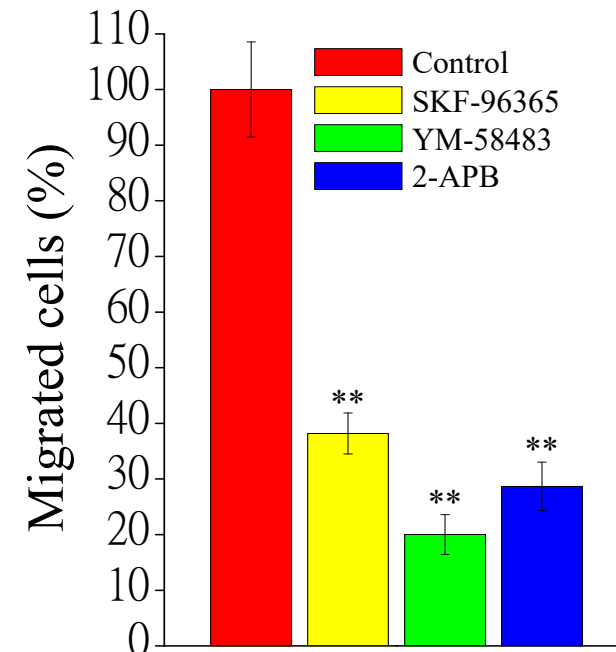**c**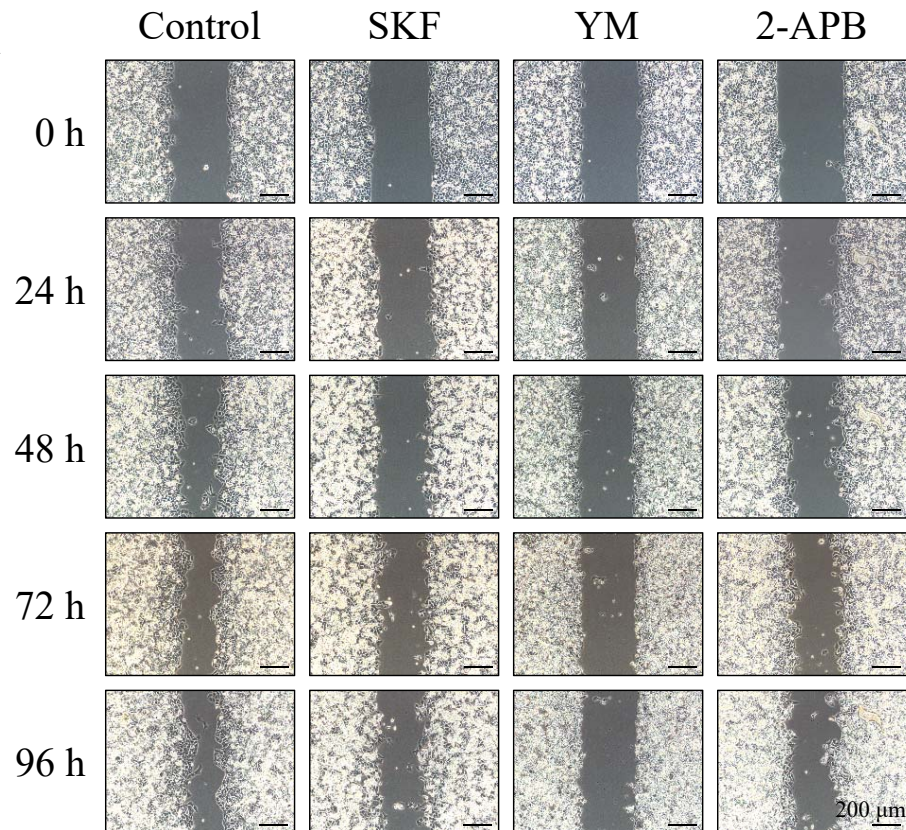**d**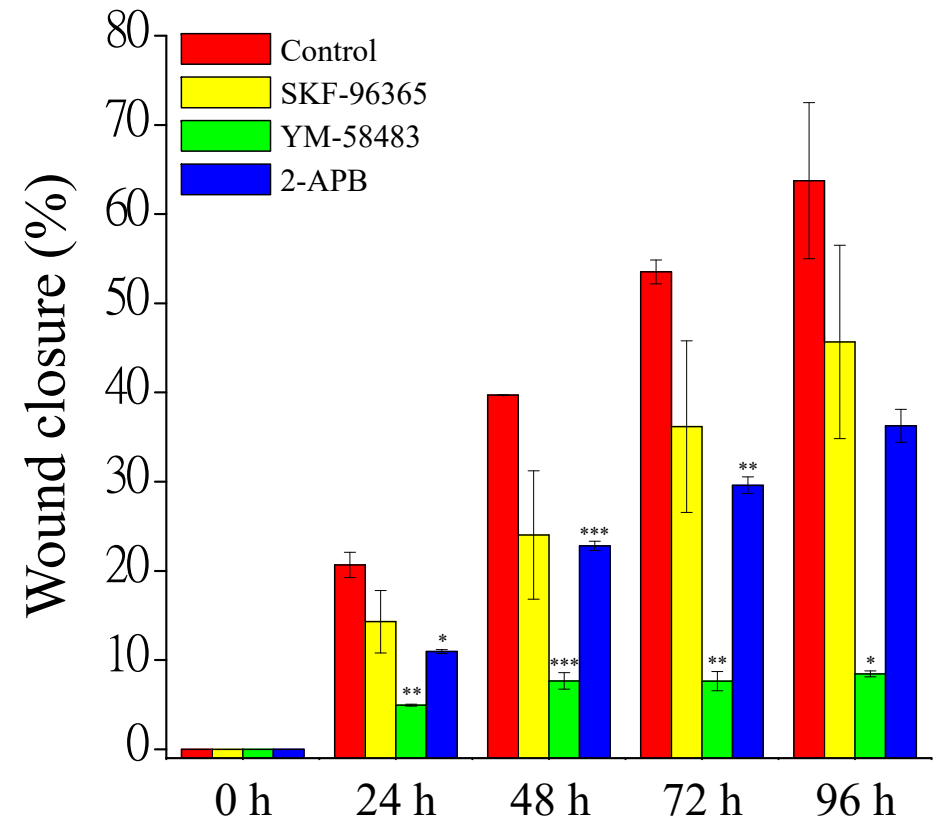

Supplement: Supplementary file 4 — Additional file 4 : Fig. S4. SOCE inhibitors decrease migration ability of IGROV1-WT cells. In vitro (a, b) transwell migration assay and (c, d) wound healing assay were performed to evaluate the effect of SOCE inhibitors (2 μΜ SKF-96365, 10 μΜ YM-58483, 0.1 μM 2-APB) on IGROV1-WT cells. a Representative photomicrographs of cells that penetrated a filter of pore size 8 μm. Scale bars, 200 μm. b Migrated cells were counted in 15 random fields on the lower surface of the filters and expressed as a percentage (%) of SOCE inhibitor pretreated cells compared with DMSO control (Ctrl). c Cells were seeded into silicon inserts with 10% FBS medium. Following cell adhesion, inserts were removed and incubated for 96 h. Phase images were captured every 24 h and wound spaces were analyzed using ImageJ. d Cellular migratory ability is presented as the percentage of wound closure. Each bar represents mean ± SEM from three independent experiments. *: significant difference between cells treated with SOCE inhibitors and DMSO control (Ctrl). *: p < 0.05; **: p < 0.01; ***: p < 0.001 by Student’s t-test. [file 12929_2020_630_MOESM4_ESM.pdf]
